# The Creation of a Novel Low-Cost Bench-Top Kidney Transplant Surgery Simulator and a Survey on Its Fidelity and Educational Utility

**DOI:** 10.7759/cureus.11427

**Published:** 2020-11-10

**Authors:** Vatche Melkonian, Tess Huy, Chintalapati R Varma, Mustafa Nazzal, Henry B Randall, Minh-Tri J Nguyen

**Affiliations:** 1 Surgery, Abdominal Transplant Center, Saint Louis University Hospital, St. Louis, USA; 2 Surgery, University of California Los Angeles, Los Angeles, USA; 3 Transplant Surgery, Transplant Institute, Loma Linda University Medical Center, Loma Linda, USA

**Keywords:** kidney transplantation, simulation training, surgical anastomosis, residency, medical education

## Abstract

Introduction

Resident inexperience during time-sensitive vascular anastomoses of a kidney transplant can negatively impact outcomes. In light of this, we created a low-cost bench-top kidney transplant surgery simulator to help residents practice vascular anastomoses.

Methods

We searched for inexpensive materials to design an iliac fossa and kidney allograft. Eighteen residents with real-life kidney transplant experience trialed the simulator and scored its fidelity and educational utility on a 0-100 visual analog scale (VAS) survey.

Results

A 35.9 x 19.4 x 12.4 cm plastic box mimicked the iliac fossa. Hooks attached to the box’s sidewall held under tension 1.27 and 0.64 cm diameter Penrose drains to replicate the external iliac vein and artery. A modified kidney-shaped stress ball with 1.27 x 4, 0.64 x 4, and 0.64 x 15 cm Penrose drains replicated a kidney allograft with its vein, artery, and ureter, respectively. Residents performed and assisted in vascular anastomoses on the simulator. The iliac fossa and allograft cost $20.20 and each practice run cost $7.20. Residents thought that the simulator was less difficult than real-life procedure, had acceptable fidelity levels, and they highly rated its educational utility.

Conclusion

Our novel low-cost bench-top kidney transplant surgery simulator focusing on vascular anastomoses received positive educational feedback from residents.

## Introduction

In the current 80-hour workweek restriction environment set by the Accreditation Council for Graduate Medical Education, the traditional Halstedian apprenticeship model of graded responsibility is likely insufficient to consistently meet the quantitative and qualitative education demands of general surgery residents while simultaneously preserving patient safety, especially during transplant surgery rotations [1]. Out of all transplant procedures, kidney transplant usually provides the best opportunity for residents to significantly put into practice vascular anastomosis, a skillset they are rarely exposed to on other surgical rotations [2]. However, residents on average participate in only 5.9 ± 7 kidney transplants during their entire training, with 30% lacking the confidence to perform vascular anastomosis by the time they graduate [3,4].

During a transplant surgery rotation, the high service demand of care for medically complex patients often negatively impacts operative room access for residents [2]. Residents also compete with transplant surgery fellows for operative volume in certain transplant centers [2]. Moreover, operative volume alone does not fully capture whether residents acquire meaningful surgical technical proficiency and procedural understanding [5]. Kidney transplants often happen on an emergent basis during off-hours, and complications are highly scrutinized with punitive actions taken against transplant centers with below-than-expected outcomes [2,6]. Attending transplant surgeons might therefore be reluctant to delegate critical portions of the case to an inexperienced resident given the time-sensitive venous and arterial anastomoses during a kidney transplant. The time to perform these anastomoses, the warm ischemic time, has in fact been shown to have a direct relationship with the development of important negative post-kidney transplant outcomes such as delayed graft function, graft loss, and mortality [7,8].

A simulation is an educational tool that is well established in non-medical fields such as the military and aviation [9]. In the general surgery setting, simulation has been incorporated into the residency curriculum as a validated tool to assess technical skills acquisition and intraoperative performance improvement in minimally invasive surgeries [10,11]. However, the availability of simulation-based tools to complement the quantitative and qualitative educational experience of residents during their transplant surgery rotation has been limited. At the national level, the American Society of Transplant Surgeons offers annual deceased human body-based simulation workshops on laparoscopic donor nephrectomy and donation after cardiac death, which is intended for transplant surgery fellows [3,12]. With regards to kidney transplant specifically, a limited number of non-validated simulation options have been described for educational purposes. Individual transplant centers have developed local pig or deceased human body wet labs for residents to learn kidney transplant [3,13,14]. Experimental three-dimensionally (3D)-printed designs of a recipient iliac fossa and kidney transplant allograft with synthetic or donor-derived iliac vessels have also been created for practicing vascular anastomosis steps of open or robotic kidney transplants [15,16]. Future validation of these kidney transplant surgery simulation options for resident education will likely be difficult due to their high cost and lack of widespread availability.

The primary objective of this study was thus to create a novel bench-top kidney transplant surgery simulator focusing on the time-sensitive vascular anastomoses, which is low-cost and easily assembled without special technical expertise or materials, for potential future use across residency training programs. The secondary objective was to survey its fidelity and educational utility among general surgery residents at our institution.

## Materials and methods

**Figure 1:**
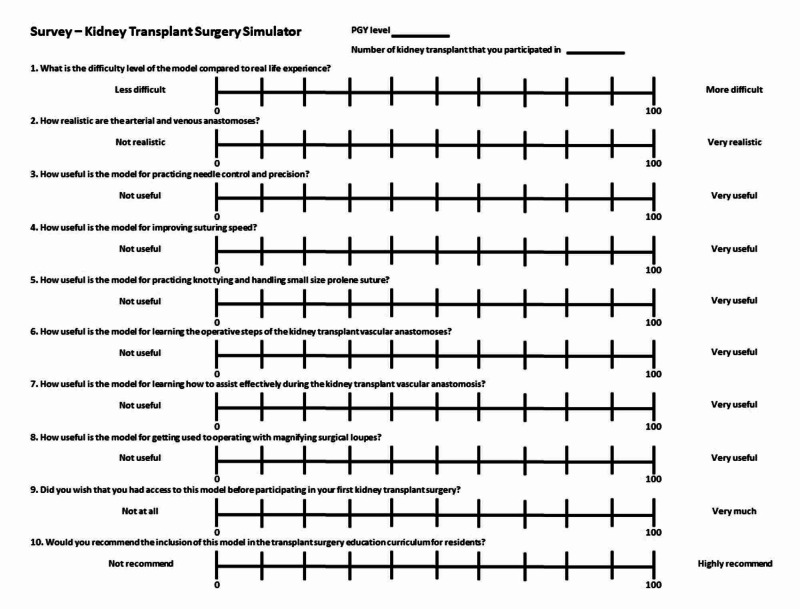
The 10-question feedback survey on the fidelity and educational utility of the novel bench-top kidney transplant surgery simulator The answers were recorded and scored using a 0-100 visual analog scale PGY: post-graduate year

This study was reviewed by the Saint Louis University Institutional Review Board and was declared exempt. In order to create a bench-top kidney transplant surgery simulator, we searched for inexpensive materials that are easily purchasable at major retail or online vendors to mimic a kidney transplant recipient’s iliac fossa with external iliac vein and artery and a kidney transplant allograft with the renal vein, renal artery, and ureter. General surgery residents from post-graduate years (PGY) 1-5 who had participated in at least one kidney transplant during their training were recruited into the study during the 2019-2020 academic year. Kidney transplant case log volume and PGY training levels were recorded for each resident. PGY 1-3 residents were classified as juniors and PGY 4-5 residents as seniors. Residents were asked to perform the operative steps associated with vascular anastomoses during real-life kidney transplants on the newly created bench-top simulator as primary surgeon and first assistant under the guidance of an attending transplant surgeon. Each resident completed a 10-question feedback survey on the fidelity and educational utility of the newly created bench-top simulator afterward. Answers to survey questions were recorded and scored using a 0-100 visual analog scale (VAS) (Figure 1). Data are expressed as mean ± standard error of the mean (SEM). Continuous variables were compared between two groups with the Mann-Whitney U test, and a p-value of ≤0.05 was considered statistically significant. Statistical analysis was performed using SPSS Statistics, version 20 (IBM, Armonk, NY).

## Results

Bench-top kidney transplant surgery simulator design

The bench-top simulator design is easy to build without the need for any special expertise and can be assembled in less than 10 minutes. Materials are easy to clean and water-resistant.

**Figure 2:**
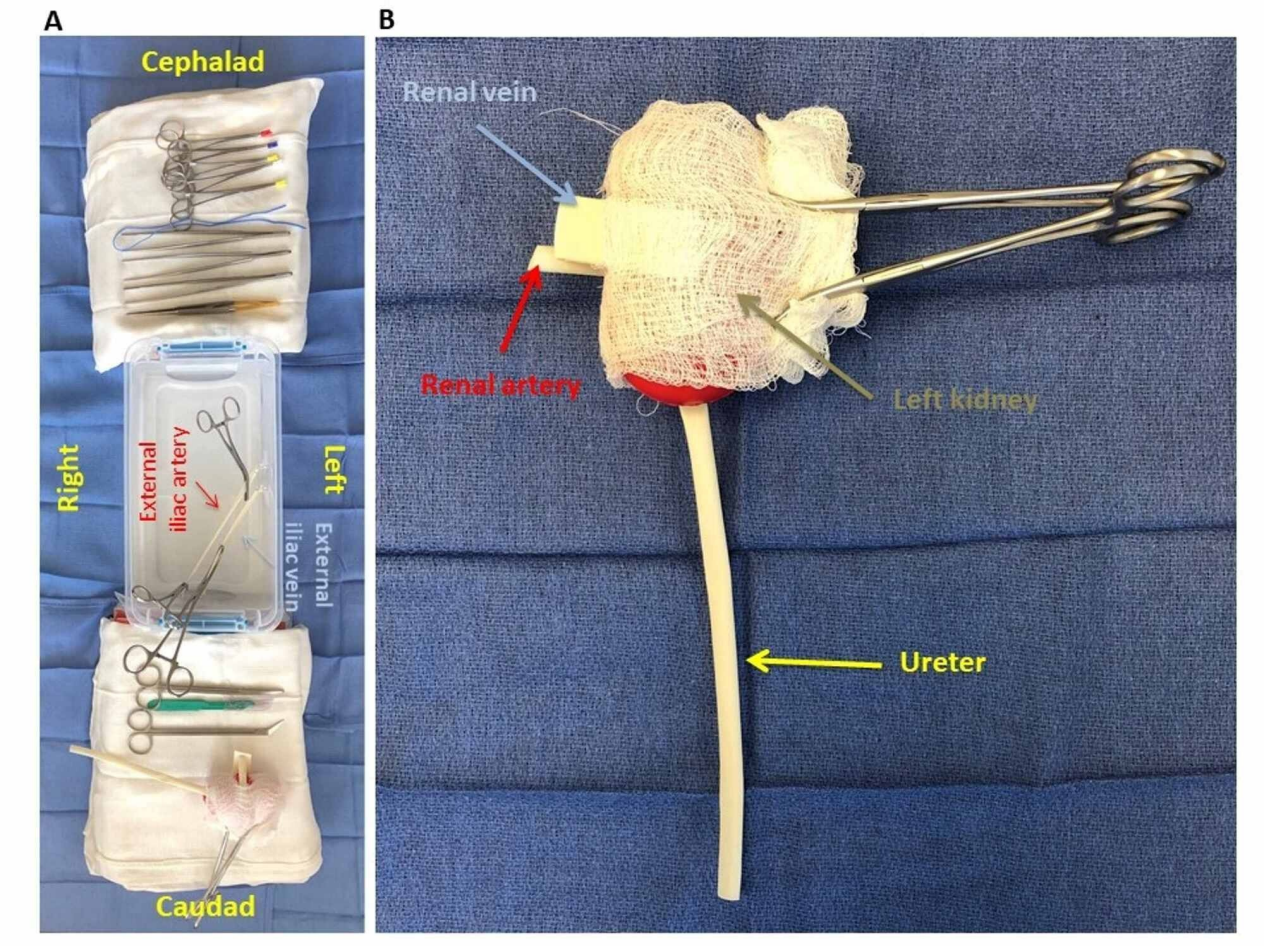
Bench-top kidney transplant surgery simulator design The images show the design replicating A) the recipient right iliac fossa and B) a left kidney transplant allograft

Kidney Transplant Recipient’s Iliac Fossa Design (Figure 2A)

A 35.9 x 19.4 x 12.4 cm (length x width x height) plastic box was used to mimic the kidney transplant recipient’s right iliac fossa (preferred laterality for kidney transplant). A total of four self-adhesive plastic mini-hooks were attached to the box’s sidewall, two side-by-side at the inferior sidewall, and two side-by-side at the medial sidewall. Penrose drains (1.27 and 0.64 cm in diameter) were cut to appropriate length to hold under tension from the plastic mini-hooks via slits created at their proximal and distal ends. The 1.27 cm diameter Penrose drain was hooked medially to replicate the external iliac vein, while the 0.64 cm diameter Penrose drain was hooked laterally to replicate the external iliac artery. The plastic mini-hooks and Penrose drains can be positioned differently inside the plastic box to construct a left iliac fossa instead if so desired.

Kidney Transplant Allograft Design (Figure 2B)

A kidney-shaped stress ball was modified by taping 1.27 x 4, 0.64 x 4, and 0.64 x 15 cm (diameter x length) Penrose drains anteriorly, posteriorly, and inferiorly to replicate a left kidney transplant allograft with its renal vein, renal artery, and ureter, respectively. The positioning of the Penrose drains can easily be modified to replicate a right allograft instead. A 10 x 10 cm gauze was held in place around the allograft with two hemostats to create a ‘jacket’ for easy-handling during vascular anastomosis. A slit was created in the gauze at the renal hilum area to leave the renal vein, renal artery, and ureter exposed.

Bench-top kidney transplant surgery simulator use

Two residents with access to surgical instruments and sutures work together on the bench-top simulator to perform the venous and arterial anastomoses operative steps of a kidney transplant (brief demonstration in Video 1). Each resident takes on the primary surgeon role for the first half of each anastomosis and the first assistant role for the other half of each anastomosis.

**Video 1:** Brief demonstration of the bench-top kidney transplant surgery simulator

Detailed Operative Steps Performed by Residents 

**Figure 3:**
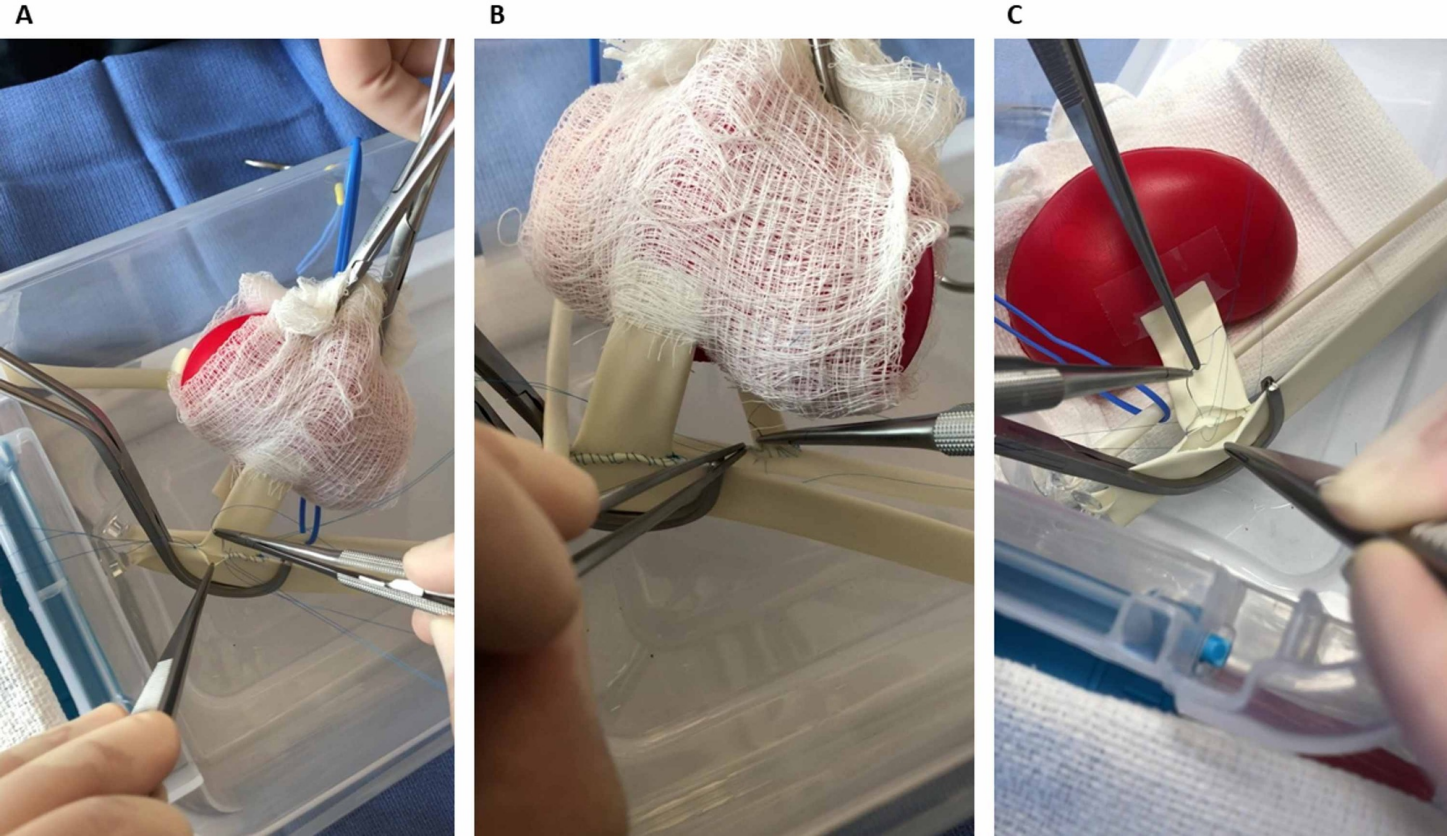
Two residents work together on the bench-top kidney transplant surgery simulator The images show residents performing the A) venous and B) arterial vascular anastomoses operative steps of a real-life kidney transplant. C) An alternative technique to the venous anastomosis is demonstrated in which the back wall is performed from inside the lumen

A vessel loop is used to retract the external iliac artery laterally. A Lambert-Kay vascular clamp is applied to the inferior aspect of the external iliac vein to obtain proximal and distal control. A venotomy is made with an 11-blade scalpel and extended to match the kidney transplant allograft renal vein diameter using 45° Potts Scissors. The kidney transplant allograft is brought to the inferior aspect of the field with its renal vein and artery oriented laterally and ureter inferiorly. An end-to-side renal vein to external iliac vein anastomosis (Figure 3A) is performed using four C1 needle double-armed 5-0 Prolene sutures. The primary surgeon holds the needle in the dominant hand on a straight Castroviejo needle holder and Gerald forceps in the non-dominant hand. The first assistant helps by using forceps, following the stitch, or handling the kidney transplant allograft. The superior and inferior corner sutures are placed first, tied down to parachute the renal vein onto the external iliac vein, and placed on rubber shods. To prevent inadvertent back wall suturing, retraction sutures are then inserted at the midpoint of the medial and lateral venous sidewalls without tying down and are placed on rubber shods. The superior stitch is sewn circumferentially around the venous anastomosis and tied at the end, with each resident performing their side and assisting on the other side. All four stitches are then cut leaving long tails. Two pediatric DeBakey vascular clamps are then applied on the external iliac artery, one inferiorly for distal control followed by one superiorly for proximal control. A site proximal and unhindered by the Lambert-Kay venous vascular clamp is chosen to create an arteriotomy with an 11-blade scalpel and extended to match the kidney transplant allograft renal artery diameter using 45° Potts Scissors. An end-to-side renal artery to external iliac artery anastomosis (Figure 3B) is performed using two C1 needle double-armed 6-0 Prolene sutures. Superior and inferior corner sutures are placed, and only the superior side is tied down. The corner sutures are placed on rubber shods. The superior stitch is sewn circumferentially around the arterial anastomosis and tied at the end, with each resident performing their side and assisting on the other side. The inferior corner suture is tied, and the two stitches are cut leaving long tails. The vascular clamps are removed in the following order: the Lambert-Kay venous vascular clamp, inferior pediatric DeBakey arterial vascular clamp, and finally the superior pediatric DeBakey arterial vascular clamp.

The operative steps described above are based on the authors’ personal technical preferences. The bench-top simulator can easily be personalized to reflect technical variations commonly used by other transplant surgeons. For example, an alternative technique to the end-to-side renal vein to external iliac vein anastomosis is to perform the back wall from inside the lumen followed by completion of the anterior wall (Figure 3C).

For repeated use of the bench-top simulator, the only replacement parts are the Penrose drains replicating the external iliac vein, external iliac artery, renal vein, and renal artery. It requires less than two minutes to replace those parts between uses and does not need any special expertise. All surgical instruments, sutures as well as reusable and disposable materials fit inside the plastic box for easy storage and transport.

Bench-top kidney transplant surgery simulator cost

The cost of building the foundation of the bench-top simulator (iliac fossa and kidney transplant allograft) is $20.20 (Table 1). The disposable cost for each use of the simulator is $7.20, accounting for the price of a 1.27 x 45.72 cm (diameter x length) Penrose drain, a 0.64 x 45.72 cm (diameter x length) Penrose drain, four expired C1 needle double-armed 5-0 Prolene sutures, and two expired C1 needle double-armed 6-0 Prolene sutures (Table 1). Reusable surgical instruments and accessories can be borrowed for free from the local institution’s operating room or purchased from online vendors for $108.10 (Table 1).

**Table 1:** Cost of the bench-top kidney transplant surgery simulator ^a^Suture prices reflect the purchase of expired products from online vendors; ^b^surgical instruments and accessories were purchased from online vendors but can alternatively be borrowed for free from the operating room

	Quantity	Cost/unit ($)	Total cost ($)
Bench-top kidney transplant surgery simulator materials			
Reusables			
Plastic box	1	5.00	5.00
Plastic mini-hooks	4	1.00	4.00
Kidney stress ball	1	10.00	10.00
Disposables			
Penrose drain 1.27 x 45.72 cm	1	0.60	0.60
Penrose drain 0.64 x 45.72 cm	1	0.60	0.60
Total	8		20.20
Sutures			
Disposables			
5-0 Prolene suture with double-armed C1 needle (91 cm)	4	1.00	4.00
6-0 Prolene suture with double-armed C1 needle (76 cm)	2	1.00	2.00
Total	6		6.00^a^
Surgical instruments and accessories			
Reusables			
Castroviejo straight needle holder (17.78 cm)	1	10.00	10.00
Lambert-Kay vascular clamp (21.59 cm)	1	20.00	20.00
DeBakey angled vascular clamp (12.7 cm)	2	10.00	20.00
Gerald tissue forceps (20.32 cm)	3	5.00	15.00
Metzenbaum curved scissors (17.78 cm)	1	5.00	5.00
Potts 45° Scissors (19.05 cm)	1	5.00	5.00
Hemostat curved clamp (12.7 cm)	5	5.00	25.00
Rubber-shod clamp cover	4	1.00	4.00
Vessel loop	1	3.00	3.00
Scalpel with 11 blade	1	1.00	1.00
Gauze sponge (10 x 10 cm)	1	0.10	0.10
Total	21		108.10^b^

Feedback survey on the fidelity and educational utility of the bench-top kidney transplant surgery simulator

**Figure 4:**
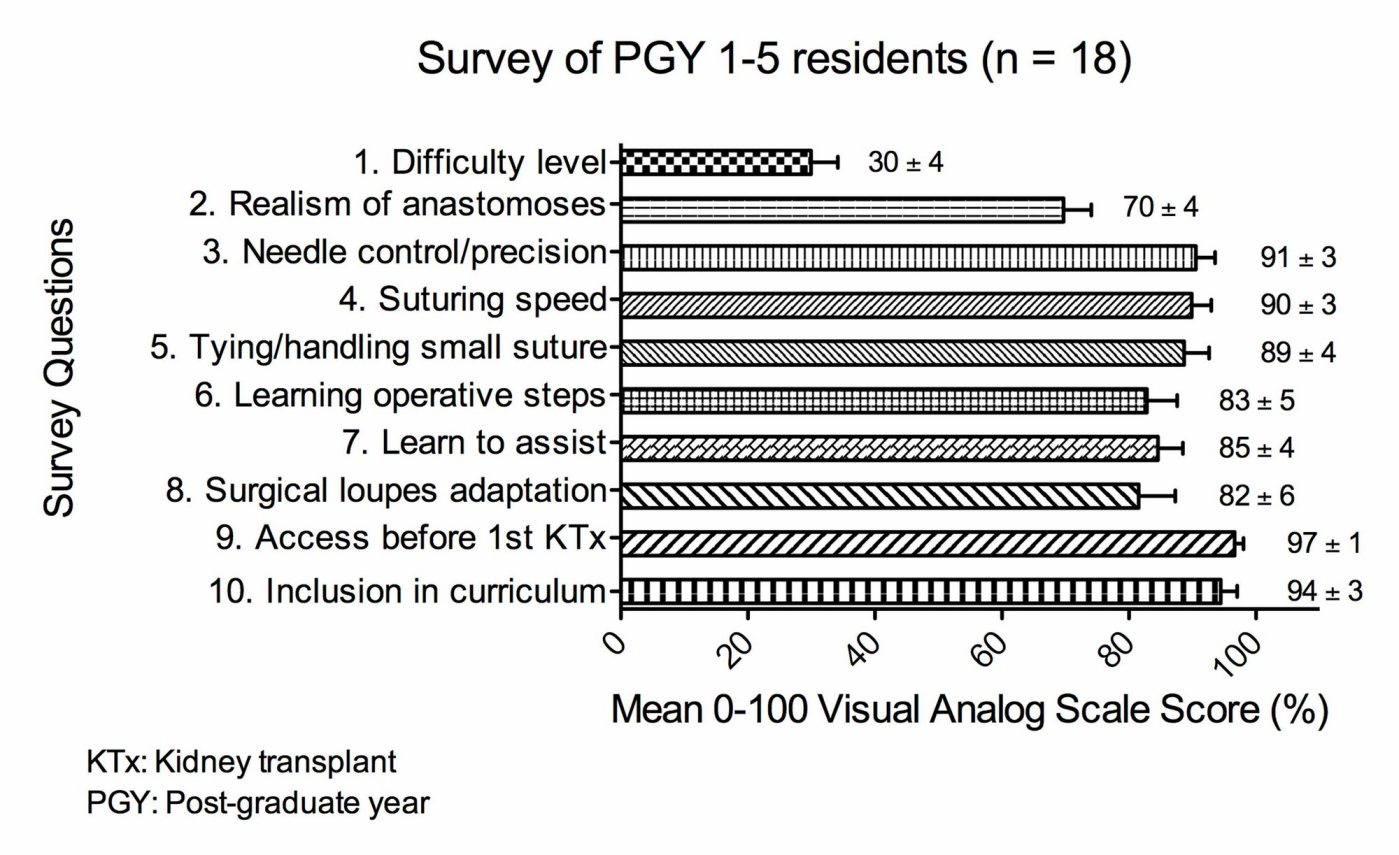
Mean 0-100 visual analog scale scores provided by PGY 1-5 general surgery residents for survey questions on the fidelity and educational utility of the novel bench-top kidney transplant surgery simulator

Eighteen PGY 1-5 general surgery residents with a mean kidney transplant case log of 9 ± 3 trialed the bench-top kidney transplant surgery simulator and completed the feedback survey on its fidelity and educational utility (Figure 4). Residents viewed the difficulty level of the bench-top simulator as easier than their intraoperative experience (mean score for question 1 = 30 ± 4%). Residents scored the simulation of the venous and arterial anastomoses as acceptably realistic (mean score for question 2 = 70 ± 4%). The simulator was highly rated for improving open vascular surgical technical skills, helping to learn the operative steps, helping to learn how to assist effectively, and aiding in getting used to magnifying surgical loupes (mean score range for questions 3-8 = 82-91%). Residents strongly wished that they had access to the simulator before participating in their first kidney transplant (mean score for question 9 = 97 ± 1%) and strongly recommended its inclusion in the transplant surgery educational curriculum (mean score for question 10 = 94 ± 3%). As expected, senior PGY 4-5 residents had a statistically higher (p: <0.01) kidney transplant case log (18 ± 4) than junior PGY 1-3 residents (2 ± 0) prior to trialing the bench-top simulator. A sub-analysis comparing more experienced senior PGY 4-5 residents with less experienced junior PGY 1-3 residents did not reveal any statistically significant differences in their scoring for the feedback survey questions (Table 2).

**Table 2:** Comparisons of kidney transplant case log number and mean visual analog scale score for survey questions regarding the bench-top kidney transplant surgery simulator between PGY 1-3 and PGY 4-5 residents ^a^Mann-Whitney U test PGY: post-graduate year

Survey questions	Mean score		P-value^a^
	PGY 1-3 (n = 10)	PGY 4-5 (n = 8)	
Case log number	2 ± 0	18 ± 4	<0.01
1. Difficulty level of the model	25 ± 6	37 ± 5	0.37
2. Realism of arterial and venous anastomoses	70 ± 5	70 ± 8	0.86
3. Practice needle control/precision	89 ± 5	93 ± 2	0.82
4. Improve suturing speed	87 ± 5	94 ± 2	0.52
5. Practice knot tying/handling small sutures	91 ± 3	86 ± 8	1.00
6. Learn operative steps	79 ± 8	88 ± 3	0.68
7. Learn to assist	87 ± 5	82 ± 6	0.50
8. Adaptation to magnifying surgical loupes	81 ± 8	83 ± 9	0.96
9. Access to model before the first kidney transplant	96 ± 2	98 ± 2	0.73
10. Inclusion of model in transplant curriculum	91 ± 4	99 ± 1	0.16

## Discussion

Residents currently graduate with a low kidney transplant case log volume at the national level [3]. It is also inconsistent as to how much they participate and learn in each case given the off-hour emergent nature of the procedure and high scrutiny on transplant outcomes [2,5,6]. We created a novel low-cost bench-top kidney transplant surgery simulator that received positive feedback from general surgery residents regarding its potential to complement their surgical educational experience during their transplant surgery rotation without impacting patient outcomes. Our novel bench-top simulator offers a controlled environment for residents to gain confidence in the time-sensitive vascular anastomoses of kidney transplants without jeopardizing patient safety via repeated practice under the mentorship of an attending transplant surgeon experienced in the procedure. The low-cost, portability, and ‘do it yourself’ assembly of the simulator allow for its widespread implementation across residency training programs and even for independent practice at home.

Although 3D-printed simulators replicating the iliac fossa and kidney transplant allograft have been described in the practice of open or robotic vascular anastomoses of kidney transplants, their higher cosmetic fidelity likely offers little educational advantages compared to our bench-top simulator, as mimicking the critical steps of a procedure rather than fidelity level is most important in skill acquisition [9,15,16]. Moreover, creating a high cosmetic fidelity replicate of the iliac fossa and allograft costs approximately $1,000 more without added education benefits such as obtaining exposure, placing retractors, or dissection [15]. As opposed to the easy assembly of our bench-top simulator, the production of a 3D-printed simulator requires approximately seven days of labor and technical expertise for imaging reconstruction from thin-cut CT imaging, working the printer, and choosing adequate ink [16]. A 3D-printed ‘hybrid’ simulator has also been described using expired deceased donor iliac vessels instead of synthetic material to simulate vascular anastomosis [15]. Although more realistic, the disadvantages of expired deceased donor iliac vessels include a limited supply and the risk of infection transmission despite testing.

Another educational strategy described to teach kidney transplants is the use of pig or Thiel’s embalmed deceased human body wet labs [3,13,14]. Compared to our bench-top simulator, which focuses solely on vascular anastomoses, pig and deceased human body wet labs offer the advantage of simulating the entire kidney transplant process with high fidelity, including donor surgery, backbench preparation of allograft, and entire kidney transplant surgery including exposure, retraction, tissue handling, dissection, vascular anastomoses, and ureteroneocystostomy. Pigs, but not deceased human bodies, also allow for handling of bleeding during dissection and reperfusion. Nevertheless, our bench-top simulator has several advantages for resident education despite its lower fidelity. Primarily, the cost of our simulator ($134.30 with and $20.20 without sutures or surgical instruments) is only a fraction of the one associated with organizing a pig or deceased human body wet lab. For example, a single pig wet lab session for six residents costs approximately $4,000 [3]. Secondly, our simulator can be used repeatedly for training multiple residents at a low cost ($7.20 per practice run), while each pig or deceased human body is single-use. Thirdly, our simulator is extremely easy to build using commercially available materials and does not require any special expertise for assembly. On the contrary, pig wet labs require an initial investment in an animal facility and veterinarians to care for the animals during pre-, intra-, and post-simulation [14]. Similarly, deceased human body wet labs require an initial investment in an anatomy facility with the expertise to perform Thiel’s embalming technique, a process that takes over six months [13]. Fourthly, since our simulator is portable, it is easily accessible to residents for training even at home. This is in contrast with pig and deceased human body wet lab sessions that require planning at a dedicated facility. Finally, our simulator is naturally exempt from the ethical and legal constraints of using pigs or deceased human bodies [17,18].

According to our survey on the fidelity and educational utility of our bench-top simulator, general surgery residents at our institution thought that it was easier than their real-life operative experience. Our simulator was purposefully made easy so that residents can focus on learning suturing, assisting, and operative steps. In the future, the difficulty level of our simulator can easily be made harder by deepening the plastic box to mimic an obese iliac fossa, increasing the kidney transplant allograft size, or shortening the renal vessels on the allograft. Despite having a higher kidney transplant case log than the national average, residents at our institution still rated the educational utility of the simulator highly and strongly recommended its inclusion in our local transplant surgery educational curriculum.

Prior to widespread implementation across residency training programs, it will be important to evaluate whether our bench-top simulator enhances the performance metrics of residents. Due to its low cost for repeated use, portability, and ‘do it yourself’ assembly, our simulator would be ideal for future multicenter studies to establish proficiency-based targets and evaluate whether skills acquired on the simulator translates into better competency during real-life kidney transplants. Based on existing low-fidelity bench-top simulators for carotid endarterectomy and gastrointestinal anastomosis, our bench-top kidney transplant surgery simulator will likely be ideal to shorten the learning curve of junior residents but can also positively impact skill acquisition at a higher level of training [19,20]. For example, senior residents and even transplant surgery fellows could demonstrate their competency in taking a junior resident through the operation on the simulator prior to gaining full independence from attending surgeons during real-life kidney transplants.

Preliminary data relating to this study were presented as an abstract at the 2020 American Society of Transplant Surgeons 20th Annual State of the Art Winter Symposium (January 9-12, Miami, FL) and at the 2020 American Transplant Congress (May 30-June 1, virtual).

## Conclusions

We created a novel bench-top kidney transplant surgery simulator focusing on the vascular anastomoses, which is low-cost, portable, and easy to assemble using widely available materials. We believe its use can potentially complement the education of residents during their transplant surgery rotation. Future validation of our simulator and its ability for performance evaluation will be required prior to its widespread adoption by residency training programs with transplant surgery rotations.
